# Muscle Weakness in an Adult With 22q11.2 Deletion Syndrome

**DOI:** 10.1111/cns.70094

**Published:** 2024-11-04

**Authors:** Cong‐Cong Wang, Yang Zhou, Xiao‐Li Li, Tong Du, Rui‐Sheng Duan

**Affiliations:** ^1^ Department of Neurology The First Affiliated Hospital of Shandong First Medical University & Shandong Provincial Qianfoshan Hospital Jinan Shandong China; ^2^ Shandong Institute of Neuroimmunology Jinan Shandong China

**Keywords:** 22q11.2 deletion syndrome, coenzyme Q10, metabolome, muscle weakness

## Abstract

This case report provides the first evidence that coenzyme Q10 may improve muscle weakness in patients with 22q11.2DS. The patient's genetic copy number deletion mutation region mainly contains *COMT*, *PRODH* functional genes related with mitochondria dynamics. The level of L‐arginine was significantly increased after treatment by coenzyme Q10 in serum.

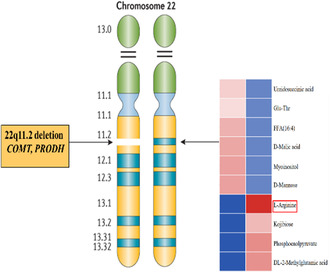

## 1 Introduction

22q11.2 deletion syndrome (22q11.2DS) is the most common human microdeletion disorder, resulting from a hemizygous 1.5–3 Mb deletion on chromosome 22q11.2 [1]. This syndrome may present with a wide spectrum of clinical manifestations, including cleft palate, heart defects, distinctive facial features, feeding difficulties, kidney abnormalities, hypoparathyroidism, hearing loss, developmental delays, certain autoimmune diseases, and learning disabilities [2]. Patients with 22q11.2DS often experience exercise intolerance, potentially linked to mitochondrial dysfunction [3, 4]. Here, we present the case of muscle weakness associated with 22q11.2DS, and explore potential therapeutic targets as well as underlying mechanisms.

## 2 Case Report

We observed a 39‐year‐old women presented with a 1‐year history of progressive muscle weakness, exercise intolerance. One year ago, the patient presented with sudden onset of limb weakness without any apparent cause. She felt difficulty in climbing stairs, experienced rapid fatigue, and was unable to walk for extended periods. The limb weakness gradually worsened, she had difficulty squatting and combing her hair, and could not raise her head when lying flat. There was no morning‐worse‐than‐evening fluctuation, no muscle twitching, muscle pain, or numbness in the limbs. The patient had no previous medical record of diabetes, metabolic issues, toxic exposures, or any neurologic illnesses. Neurologic examination revealed bilateral upper limbs weakness (4/5 shoulder abduction and 5/5 fingers abduction), bilateral lower limbs weakness (4/5 knee flexion, 4/5 knee extension, 5/5 dorsiflexion, and 5/5 toe extension) (Medical Research Council). Both limbs display symmetrical musculature with well‐maintained muscle tone, showing no signs of atrophy or rigidity.

Initially misdiagnosed with myasthenia gravis, she failed to respond to treatment with methylprednisolone and pyridostigmine bromide. Brain MRI revealed no abnormalities. Routine blood tests, electrolyte levels, and muscle enzyme measurements were within normal limits. Thyroid ultrasound showed multiple nodules, but thyroid hormone levels were normal. Electrocardiogram, cardiac ultrasound, and chest CT scans did not show significant abnormalities. The results of the electromyography repetitive nerve stimulation test were also found to be negative. Antibodies to acetylcholine receptors (AChR), muscle‐specific kinase, and lipoprotein receptor‐related protein 4 were absent. Given the patient's exercise intolerance and short stature, whole genome sequencing was performed to rule out mitochondrial encephalomyopathy or other genetic disorders. Whole genome sequencing revealed a 1.59 Mb deletion on chromosome 22q11.2, with the precise breakpoint coordinates identified as 18752780_20345438del. This deletion involves a proximal nested LCR22A‐LCR22B deletion, thereby confirming the diagnosis of 22q11.2DS. The deleted region includes pathogenic genes such as *TBX1*, *COMT*, and *PRODH*.

As there is no specific treatment for 22q11.2DS, management focuses on addressing individual symptoms. Given the genetic analysis revealed a copy number deletion encompassing the *COMT* and *PRODH* genes, both linked to mitochondrial dynamics. Therefore, coenzyme Q10 (30 mg/day) for 6 months was administered to the patient, which is commonly used in mitochondrial myopathies [5]. After treatment, the symptoms of muscle weakness gradually improved. She was capable of squatting and combing her hair with ease, and the ability to raise the head while lying down was restored. Her walking ability had improved significantly as measured by the 6‐min walk test (6MWT). The 6MWT was widely utilized for assessing exercise tolerance, the impact of medical interventions, and disease prognosis due to its practicality and effectiveness. Numerous studies have indicated that the 6MWT for healthy adults typically ranges from 400 to 700 m. The 6‐min walk test distance increased to 560 m from < 100 m previously. Neurologic examination revealed that the muscle strength of all four limbs was at grade 5.

## 3 Metabolomics Analysis

**FIGURE 1:**
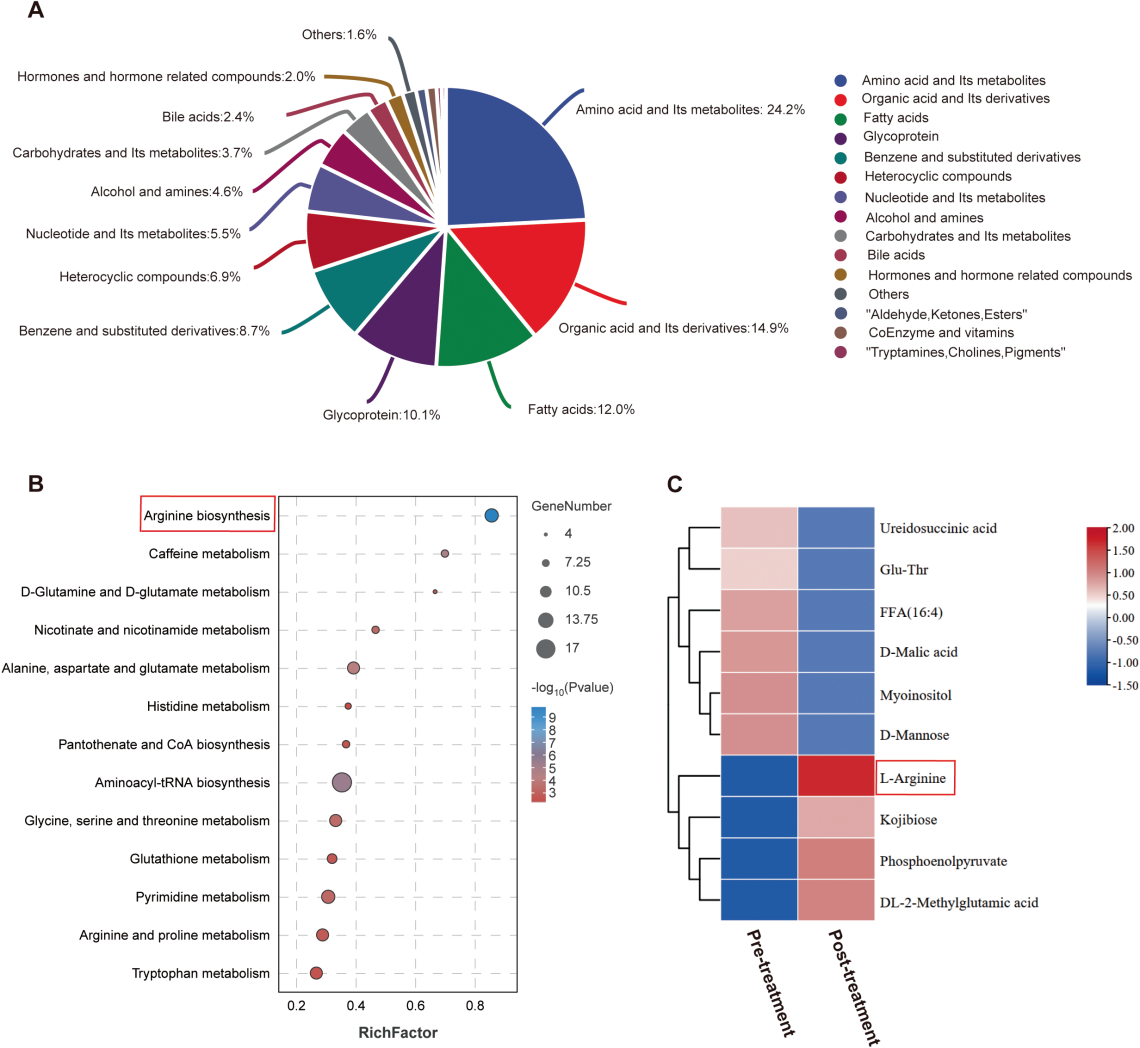
Serum metabolite profile of the patient before and after treatment. (A) Principal composition changes of serum metabolites before and after treatment. (B) The most significantly changed metabolite pathways in the patient before and after treatment. (C) Cluster analysis of top 10 metabolites in the patient compared with before treatment.

To explore the effect of coenzyme Q10 treatment on metabolic pathways in 22q11.2DS, we performed targeted serum metabolomics analysis encompassing diverse biosynthetic pathways both before and after treatment. The serum metabolome underwent substantial changes following 6 months of coenzyme Q10 supplementation, as revealed by clustering analysis. A detailed examination of the 330 metabolites identified amino acids and their derivatives (24.2%), organic acids and their derivatives (14.9%), and fatty acids (12.0%) as the primary contributors to these metabolic shifts (Figure 1A). Among these, arginine biosynthesis emerged as a significantly impacted pathway (Figure 1B). After administering coenzyme Q10, the level of L‐arginine, essential for nitric oxide‐associated muscle contraction and mitochondrial oxidative phosphorylation and functions in improving muscle performance, was significantly increased (Figure 1C).

## 4 Discussion

This case report provides the first evidence that coenzyme Q10 may improve muscle weakness in patients with 22q11.2DS. While 22q11.2DS is primarily linked to a 3‐Mb deletion on chromosome 22q11.2 composed of low copy number repeats divided into regions A–D, the most common deletion type, affecting 85% of patients, spans regions A–D and includes the *TBX1* gene, which is believed to be associated with the syndrome's characteristic clinical features, particularly cardiac anomalies. Nine (*COMT*, *UFD1L*, *DGCR8*, *MRPL40*, *PRODH*, *SLC25A1*, *TXNRD2*, *T10*, and *ZDHHC8*) of the approximately 30 genes in this region have the potential to disrupt mitochondrial metabolism. Among these genes, it is believed that the initial three exert an indirect influence on mitochondrial functioning, whereas the latter six participate directly in the operation of mitochondria [6]. Previous studies have reported metabolic abnormalities in 22q11.2DS [7], including decreased ATP levels due to impaired oxidative phosphorylation and increased glycolysis with elevated levels of 2‐hydroxyglutarate, fatty acids, and cholesterol [8].

Our patient presented with exercise intolerance resembling mitochondrial myopathy. Interestingly, coenzyme Q10 led to a remarkable improvement of muscle weakness and exercise intolerance. Coenzyme Q10 is essential for ATP production within mitochondria, functioning as an electron carrier between respiratory complexes. Its lipid solubility facilitates movement across the mitochondrial inner membrane. It relies on electron transport chains to complete energy conversion and ATP formation [9]. Additionally, it is an open label trial, and that there was no rechallenge, lifestyle factors including balanced nutrition, moderate exercise, and emotional stability, can also impact the prognosis of muscle weakness.

While 22q11.2DS exhibits diverse clinical manifestations, mitochondrial myopathy‐like features are uncommon. This patient has excluded anemia, hypocalcemia, and hypomagnesemia through the completion of blood routine and electrolyte examinations. Despite a normal chest CT, further examinations are still required to rule out asthma and obstructive sleep apnea. By examining metabolic characteristics in 22q11.2DS compared with before treatment, we aimed to elucidate biological mechanisms underlying the phenotype of muscle weakness and exercise intolerance and promote the identification of novel therapeutic targets. However, it is important to note that this is a single case report, and further research is needed to establish an internal relationship and identify additional factors influencing treatment response.

## Ethics Statement

The study was approved by the ethics committee of Shandong Provincial Qianfoshan Hospital (no. S004).

## Consent

We have obtained the patient's permission and informed consent for the publishing of his information and images.

## Conflicts of Interest

The authors declare no conflicts of interest.

## Data Availability

The data that support the findings of this study are available from the corresponding author upon reasonable request.
